# Organocatalytic tandem Michael addition reactions: A powerful access to the enantioselective synthesis of functionalized chromenes, thiochromenes and 1,2-dihydroquinolines

**DOI:** 10.3762/bjoc.8.191

**Published:** 2012-10-04

**Authors:** Chittaranjan Bhanja, Satyaban Jena, Sabita Nayak, Seetaram Mohapatra

**Affiliations:** 1 Department of Chemistry, Utkal University, Bhubaneswar-751 004, Odisha, India; 2 Department of Chemistry, Ravenshaw University, Cuttack-753 003, Odisha, India, Fax: +91-671-2610304

**Keywords:** chromenes, 1,2-dihydroquinolines, enantioselective, Michael addition, organocatalytic, thiochromenes

## Abstract

Enantioselective organocatalysis has become a field of central importance within asymmetric chemical synthesis and appears to be efficient approach toward the construction of complex chiral molecules from simple achiral materials in one-pot transformations under mild conditions with high stereocontrol. This review addresses the most significant synthetic methods reported on chiral-amine-catalyzed tandem Michael conjugate addition of heteroatom-centered nucleophiles to α,β-unsaturated compounds followed by cyclization reactions for the enantioselective construction of functionalized chiral chromenes, thiochromenes and 1,2-dihydroquinolines in optically enriched forms found in a myriad of bioactive natural products and synthetic compounds.

## Introduction

**Figure 1:**
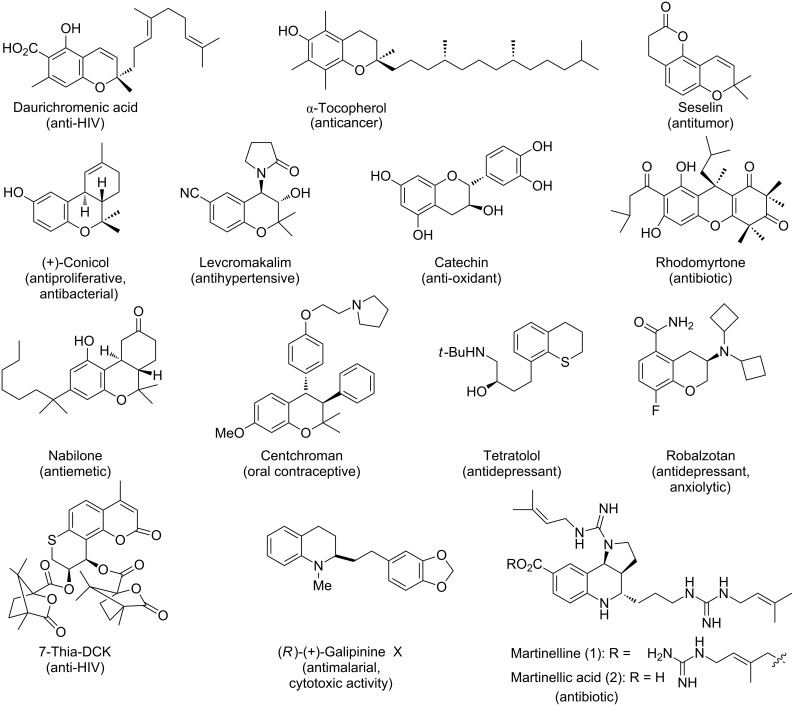
Some representative molecules having chromene, thiochromene or 1,2-dihydroquinolin structural motifs.

Chromenes or benzopyrans and their sulfur and nitrogen analogues are important classes of structural motifs found in numerous naturally occurring and synthetic compounds. Due to a rich array of functionalities and chiral centers these motifs are widely recognized as useful building blocks for the synthesis of a broad and interesting range of biologically active heterocyclic compounds having antiviral, antitumor, antimicrobial, antidiabetic, sex-pheromone, diuretic, anticoagulant, anti-anaphylatic and many more activities [1–9]. Some representative molecules of these structural motifs are shown in Figure 1 [9–21]. Therefore, synthetic methodologies allowing rapid access to these heterocycles in optically enriched form are highly desirable in organic synthesis and chemical biology/medicinal chemistry. In the past few years very promising progress has been made in this intriguing area, and among the advances, organocatalytic enantioselective methodologies have gained much attention from many research groups worldwide [22–29]. In the meantime, organocatalytic tandem Michael conjugate additions of heteroatom-centered nucleophiles to α,β-unsaturated compounds appear as one of the most reliable and powerful tools for the stereocontrolled access to a wide range of biologically active heterocycles in optically enriched form [29–32]. In this review we have summarized our efforts to cover various chiral-amine-catalyzed synthetic protocols leading to one-pot enantioselective synthesis of six membered mono hetero-atom containing, biologically active heterocycles, such as functionalized chromenes (benzopyranes), thiochromenes (thiobenzopyranes) and 1,2-dihydroquinolines, by means of tandem/domino hetero Michael addition reactions, or modified versions [33–38], covering the literature up to 2011. Keeping an overview of organocatalytic modes of activation, and taking the less reactive Michael acceptor into account, we discuss here only the iminium/enamine activation or dual activation by iminium and hydrogen-bonding interaction strategies followed by cyclization, for these one-pot enantioselective syntheses. Wherever possible, working mechanistic models are presented.

**Figure 2:**
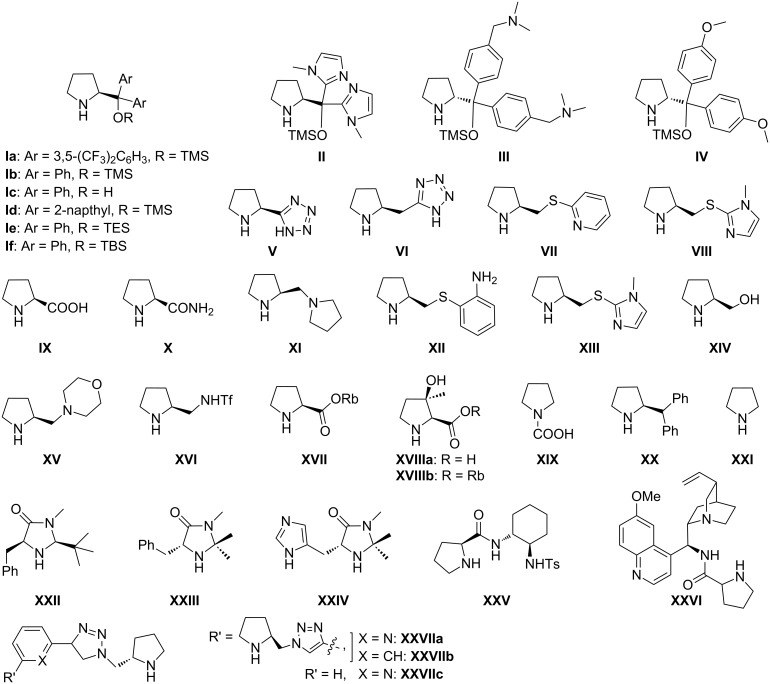
Screened chiral proline and its derivatives as organocatalysts. Rb = rubidium.

**Figure 3:**
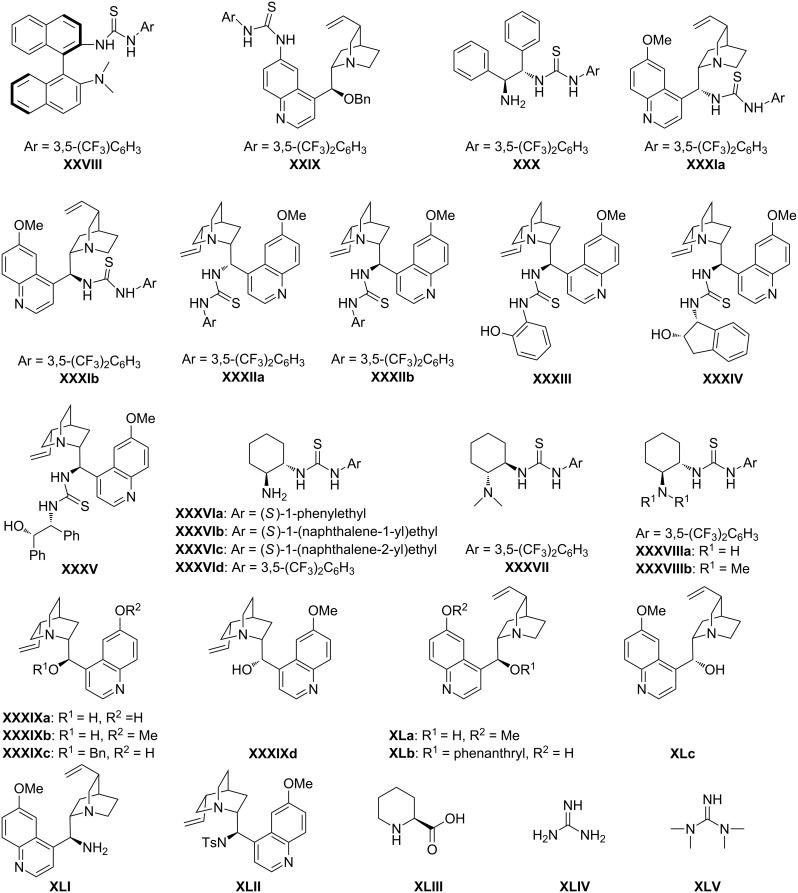
Screened chiral bifunctional thiourea, its derivatives, cinchona alkaloids and other organocatalysts.

This review work is systematized under the headings (1) Organocatalytic oxa-Michael reactions to access functionalized chromenes; (2) Organocatalytic thio-Michael reactions to access functionalized thiochromenes; and (3) Organocatalytic aza*-*Michael reactions to access functionalized 1,2-dihydroquinolines, using chiral proline and its derivatives (Figure 2), chiral bifunctional thioureas, cinchona alkaloids and other organocatalysts (Figure 3). For each reaction, the initial screening result of various organocatalysts with their percentage of conversion (% yield) and enantiomeric excess (ee) is presented in tabular form, and the best catalyst is used for the given individual scheme.

## Review

### 1 Organocatalytic oxa-Michael additions to access functionalized chromenes

#### 1.1. Reactions of 2-hydroxybenzaldehydes with acyclic/cyclic α,β-unsaturated compounds

**Scheme 1:**
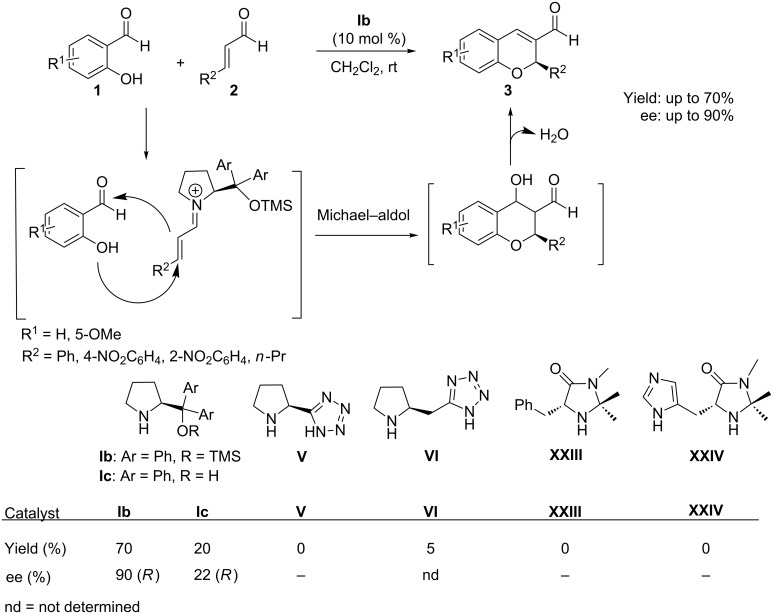
Diarylprolinolether-catalyzed tandem oxa-Michael–aldol reaction reported by Arvidsson.

The racemic synthesis of 2*H*-chromene was reported by Bräse et al. in 2005 [39–40]. A strategy based on the organocatalytic enantioselective synthesis of chiral 2*H*-chromenes through tandem-oxa-Michael–aldol sequence was first reported by Arvidsson et al. [41] in 2006, using diarylprolinolether as an effective organocatalyst. This method involved an oxa-Michael attack of salicylaldehydes **1** on the α,β-unsaturated aldehydes **2** activated through an iminium ion formation with the catalyst **Ib**, followed by an intramolecular aldol reaction and the subsequent water elimination to afford the chromene **3** (Scheme 1). The same reaction was also repeated with various catalysts, which are presented in Scheme 1. Several base and acid additives were found to affect both the enantioselectivities and the yields of the product. The overall reaction sequence provided chromenes with aromatic substituents at the C-2 position in up to 70% yield and 60% enantioselectivity in dichloromethane at room temperature, while C-2 aliphatic analogues were obtained in 90% enantiomeric excess, but with only 20% yield, under identical conditions.

**Scheme 2:**
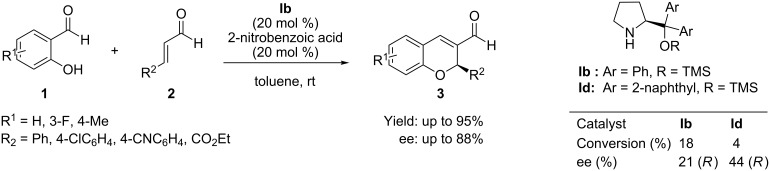
Tandem oxa-Michael–aldol reaction developed by Córdova.

Taking the advantages of the above methodology, Córdova et al. [42–43] and subsequently Wang et al. [44] independently reported similar oxa-Michael/aldol reactions by means of the same iminium-ion activation strategies but with improved yields and enantioselectivities. Córdova et al. reported the tandem reaction of salicylaldehydes **1** and α,β-unsaturated aldehydes **2** catalyzed by diphenylprolinol ether **Ib** at a slightly higher catalyst loading (20 mol %) in toluene and with 2-nitrobenzoic acid as cocatalyst, which significantly increased the ee of the reaction from 9 to 88%. Further enhancement of the yield was achieved by the use of molecular sieves (4 Å) in the reaction (Scheme 2).

**Scheme 3:**
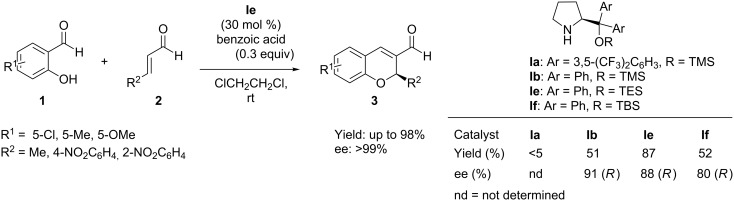
Domino oxa-Michael-aldol reaction developed by Wei and Wang.

Wang et al. [44] investigated the same tandem reaction of salicylaldehydes **1** and α,β-unsaturated aldehydes **2** employing TES-protected diphenylprolinol **Ie** as organocatalyst with high catalyst loading (30 mol %). With benzoic acid as cocatalyst and dichloroethane as solvent, the test reaction provided the chiral chromenes **3** in good yields (up to 98%) and enantioselectivities (99%) at room temperature (Scheme 3).

**Scheme 4:**
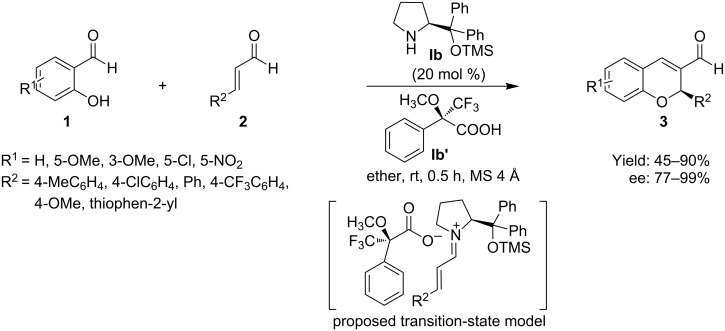
Chiral amine/chiral acid catalyzed tandem oxa-Michael–aldol reaction developed by Xu et al.

In 2009, Xu et al. [45] developed an efficient protocol for the asymmetric tandem oxa-Michael–aldol reaction using chiral amine/chiral acid organocatalyst, instead of only organocatalyst, for the enantioselective synthesis of 2*H*-chromenes. In the reported protocol, the reaction of salicylaldehydes **1** with α,β-unsaturated aldehydes **2** catalyzed by (*S*)-diphenylprolinol trimethylsilyl ether **Ib** with (*S*)-Mosher acid **Ib’** afforded the desired products **3** with high yield (45–90%) and with high enantioselectivity (77–99%) (Scheme 4). The reaction proceeded through the iminium intermediate, and the synergistic ionic interaction of chiral amine with chiral acid formed in situ in the catalytic system effected an improvement of the reaction performance and offered an efficient steric effect in the transformation. Although the reaction tolerated a broad scope of substrates, the yields as well as enantioselectivities were greatly affected by the electronic and steric effect of the substrates. Compound **1** bearing electron-donating groups afforded the desired product with high yield (up to 90%) and enantioselectivity (up to 99%), whereas compound **2** having electron-withdrawing groups provided poor results.

**Scheme 5:**
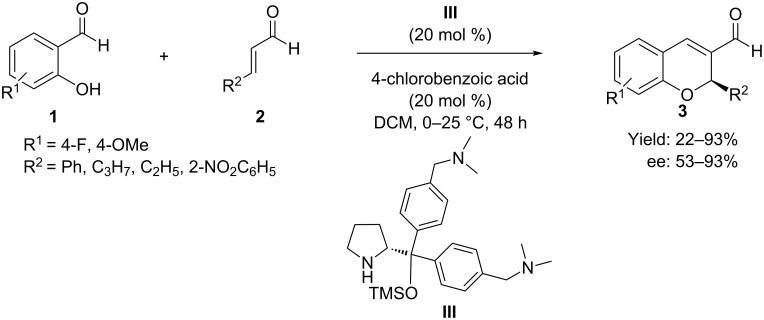
Modified diarylproline ether as amino catalyst in oxa-Michael–aldol reaction as reported by Xu and co-workers.

Very recently, Xu et al. [46] reported an improved protocol for the domino-oxa-Michael reaction of salicylaldehydes **1** with α,β-unsaturated aldehydes **2** employing tertiary amine-modified diarylpyrrolinol-TMS ether **III** as a water-soluble and recyclable organocatalyst with 4-chlorobenzoic acid as cocatalyst (Scheme 5) for the synthesis of 2*H*-chromene derivatives **3**. The electronic effect of the tertiary amine group in the modified catalyst was believed to enhance the enantioselectivity of the chiral secondary amine. Since the catalyst is soluble in water, the method provides the pure product with excellent enantioselectivity. The reaction appears to have a broad scope, but efficiencies (22–93% yield) and enantioselectivities (53–93% ee) vary with the electronic and steric nature of **1** and **2**.

**Scheme 6:**
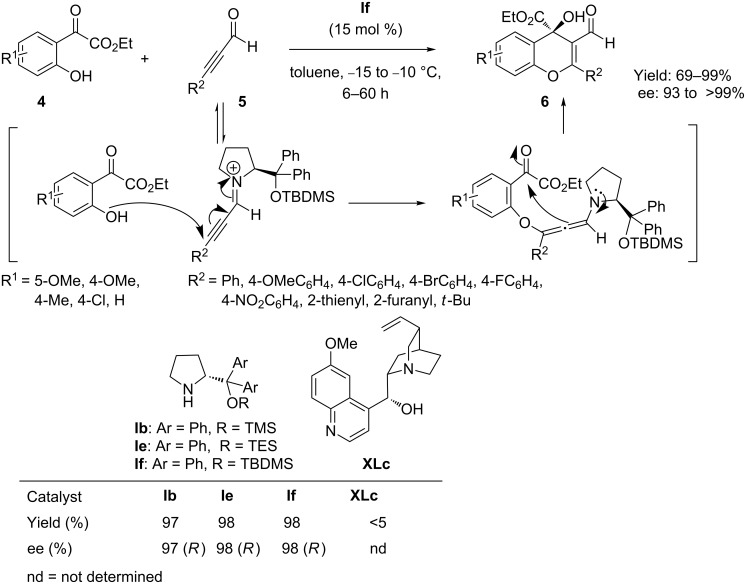
Chiral secondary amine promoted oxa-Michael–aldol cascade reactions as reported by Wang and co-workers.

A potential “one-pot” approach to the organocatalytic synthesis of chiral 4*H*-chromenes was first reported by Wang and co-workers [47]. In an unprecedented oxa-Michael–aldol cascade sequence, reactions of (2-hydroxyphenyl)-2-oxoacetates **4** (Michael donor) and alkynals **5** (Michael acceptor) afforded highly functionalized chiral 4*H*-chromenes **6** with a quaternary stereogenic center, by means of the less explored iminium-allenamine activation strategy. Among the screened chiral secondary amine catalysts, (*S*)-diphenylpyrrolinol tertiary butyldimethylsilylether **If** in toluene provides good yields and high enantiomeric excess of 4*H*-chromenes **6** with a wide range of substrates (Scheme 6).

**Scheme 7:**
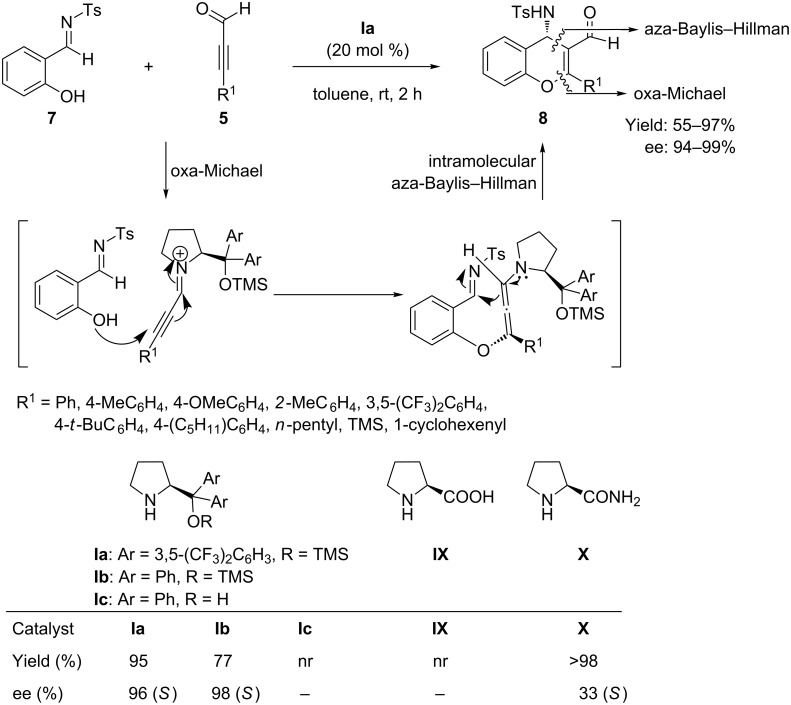
Reaction of salicyl-*N*-tosylimine with aldehydes by domino oxa-Michael/aza-Baylis–Hillman reaction, as reported by Alemán and co-workers.

The potential of less well-explored alkynals as Michael acceptors was further explored, when Alemán et al. [48] in 2010 reported the synthesis of chiral 4-amino-4*H*-chromene **8** by reaction of salicyl-*N*-tosylimine **7** (Michael donor) with 2-alkynals **5** using diarylprolinolether **Ia** as organocatalyst (Scheme 7). The reactions were carried out in toluene at room temperature and completed within 2 h, giving high yield (>97%) and excellent enantioselectivity (99%). Mechanistically the reaction proceeded through oxa-Michael addition followed by aza-Baylis–Hillman reaction by means of an iminium-allenamine activation strategy.

**Scheme 8:**
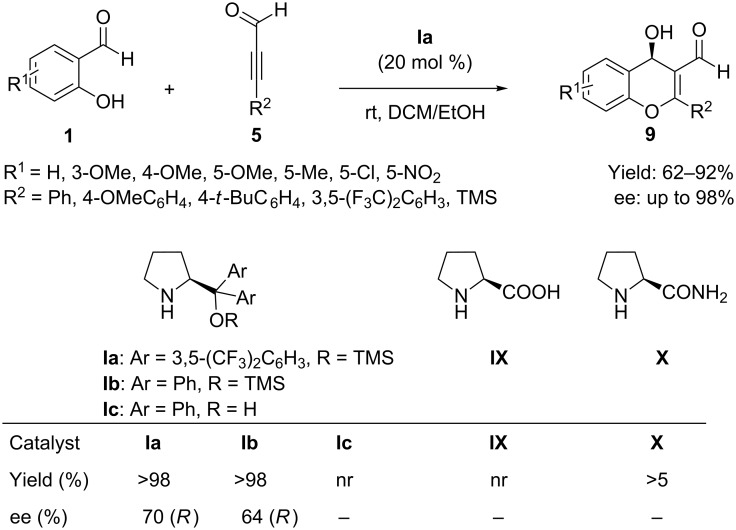
Silyl prolinol ether-catalyzed oxa-Michael–aldol tandem reaction of alkynals with salicylaldehydes reported by Alemán group.

Later on, the same authors [49] in 2011 reported a similar tandem reaction (abnormal Baylis–Hillman) between 2-alkynals **5** and salicylaldehyde derivatives **1** catalyzed by proline derivatives (prolinamide or prolinol) leading to optically active 4-hydroxy-4*H* chromene-3-carbaldehydes **9** (Scheme 8). The reactions proceeded with good yields and enantiomeric ratio up to 98:2 by using the bulky catalyst prolinol diphenyl silylether **Ia** in dichloromethane/ethanol (1:1) at room temperature.

**Scheme 9:**
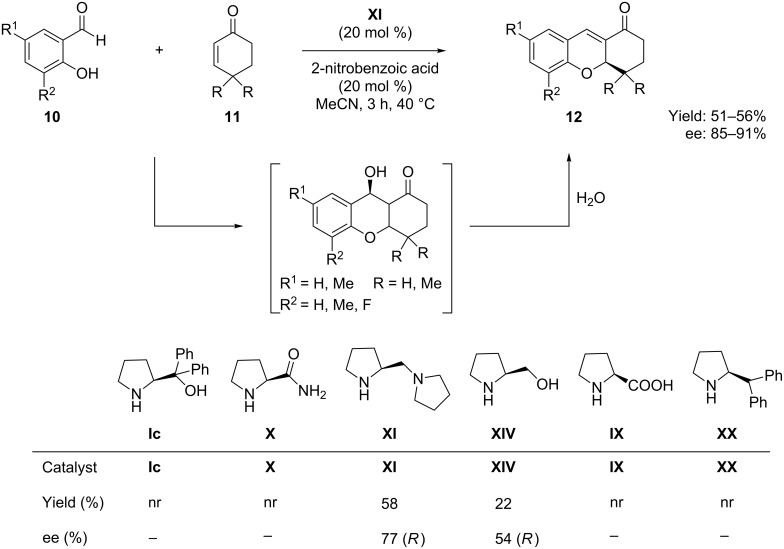
Oxa-Michael–aldol sequence for the synthesis of tetrahydroxanthones developed by Córdova.

Cyclic α,β-unsaturated ketones have also responded as Michael acceptors in the organocatalytic tandem Michael addition reaction towards the synthesis of tetrahydroxanthones. Córdova et al. [50], in 2007, reported the first organocatalytic asymmetric synthesis of tetrahydroxanthenones through the domino Michael–aldol reaction of salicylaldehyde derivatives **10** and α,β-unsaturated cyclic ketones **11**. Using chiral pyrrolidine **XI** as organocatalyst and 2-nitrobenzoic acid as additive, the reaction of salicylaldehydes and α,β-unsaturated cyclic ketones afforded the corresponding tetrahydroxanthones **12** with moderate yields (up to 56%) and enantioselectivities in the range of 85–91% ee (Scheme 9)*.* Mechanistically the reaction involves the iminium activation of the α,β-unsaturated cyclic enones by the chiral pyrrolidine catalyst.

**Scheme 10:**
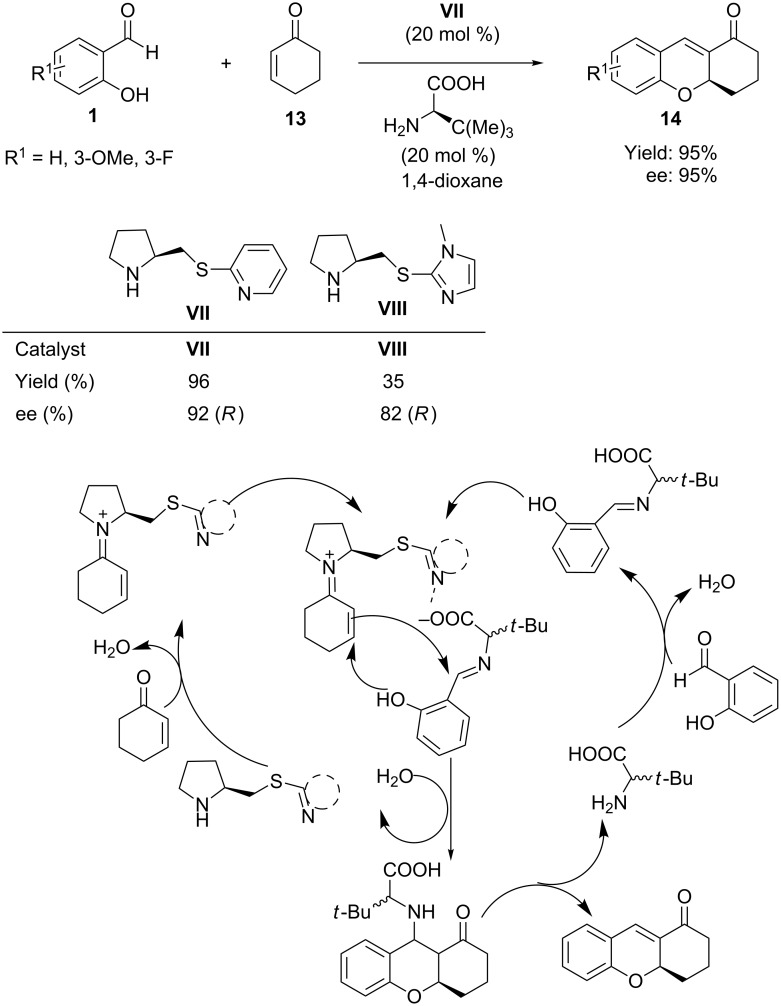
Synthesis of tetrahydroxanthones developed by Xu.

Very recently, Xu and co-workers [51] reported an improved protocol for the same reaction employing a chiral pyrrolidine bearing a 2-mercaptopyridine moiety as organocatalyst (**VII**) and simple α-amino acids, such as *tert*-leucine, as co-catalyst. The asymmetric transformation proceeds by simultaneous activation of cyclic enones **13** and aldehyde **1** by the bifunctional catalyst **VII** and the amino acid, respectively, via the generation of a transient ion pair through iminium and imine intermediates. The reaction afforded the corresponding tetrahydroxanthenones **14** in excellent yields (95%) and enantioselectivities (95%) (Scheme 10).

#### 1.2. Reaction of 2-hydroxycinnamaldehydes/2-hydroxy-β-nitrostyrene with acyclic α,β-unsaturated compounds

**Scheme 11:**
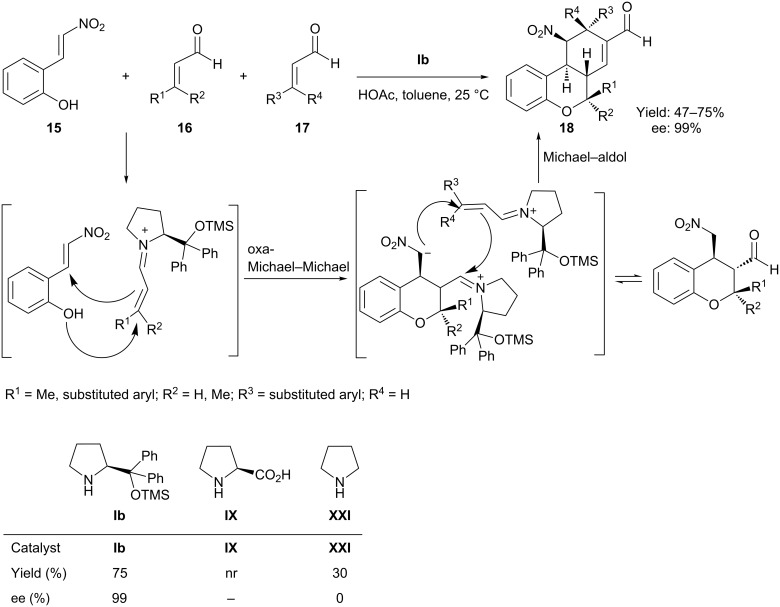
Diphenylpyrrolinol trimethylsilyl ether catalyzed oxa-Michael–Michael–Michael–aldol reaction for the highly stereogenic synthesis of chromenes.

In the previously reported organocatalytic tandem oxa-Michael–aldol reactions, the instantaneous dehydration of the β-hydroxyaldimine intermediates generates products with only one stereogenic center. Hong et al. [52] in 2009 reported a novel quadruple-cascade reaction for constructing highly functionalized and enantiomerically enriched tetrahydro-6*H*-benzo[*c*]chromenes with five stereogenic centers. The reaction involved a domino oxa-Michael–Michael–Michael–aldol condensation of *o*-hydroxy-β-nitrostyrene **15** and two equivalents of α,β-unsaturated aldehydes in the presence of diphenylpyrrolinol trimethylsilyl ether **Ib** with an acid additive (HOAc/4-nitrobenzoic acid) in toluene at ambient temperature, which afforded tetrahydro-6*H*-benzo[*c*]chromenes **18** in high diastereoselectivity and excellent enantioselectivity (>99% ee) (Scheme 11). The proposed mechanism of the cascade reaction starts in an analogous manner to that previously mentioned, although the first oxa-Michael step is followed by a second Michael addition to form the chroman unit. A series of α*,*β-unsaturated aldehydes were reacted with the *o*-hydroxy-β-nitrostyrene as shown in Scheme 11. Except for the observation of a trace of intermediate, only one enantiomer was observed in this reaction, probably due to the first oxa-Michael addition, which is known to proceed with high diastereo- and enantioselectivity, and the resulting product presumably directs the stereochemistry of the subsequent reactions. This quadruple-cascade reaction exemplifies an efficient three-component reaction.

**Scheme 12:**
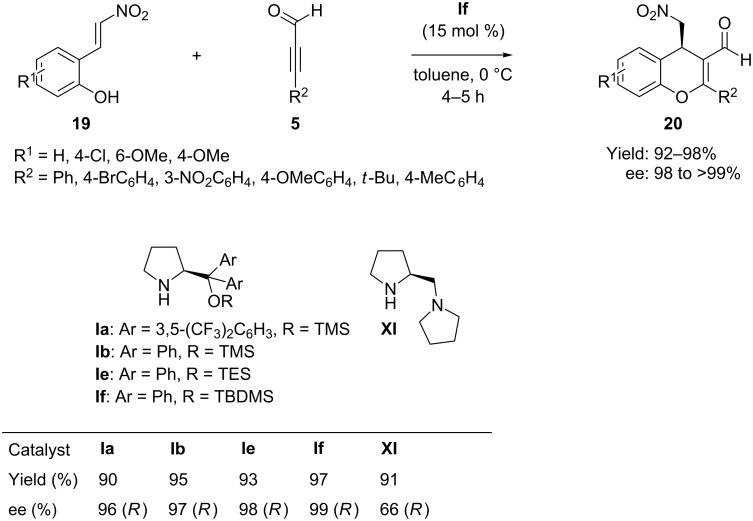
Enantioselective cascade oxa-Michael–Michael reaction of alkynals with 2-(*E*)-(2-nitrovinyl)-phenols reported by Wang.

In 2010 Wang et al. [11] reported the highly enantioselective synthesis of trisubstituted chiral 4*H*-chromenes **20** through iminium-allenamide catalysis. The reaction consists of a Michael–Michael-cascade sequence between alkynals **5** and *o*-hydroxy-β-nitrostyrenes **19**, catalyzed by diphenylprolinol TBDMS ether **If** as catalyst in toluene at 0 °C (Scheme 12). Mechanistically, alkynals were involved in an unprecedented iminium-allenamine sequence and afforded highly functionalized 4*H*-chromenes in high yields (92–98%) and with high ee (>99%).

**Scheme 13:**
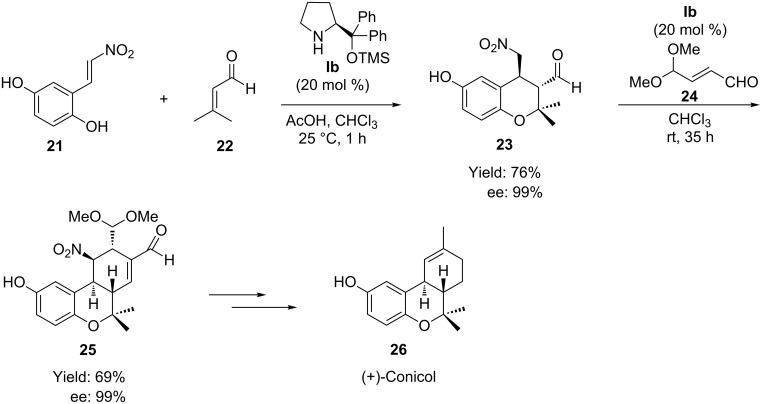
Domino oxa-Michael–Michael–Michael–aldol reaction of 2-(2-nitrovinyl)-benzene-1,4-diol with α,β-unsaturated aldehydes presented by Hong group.

Extending the methodology adopted by Hong et al., the same group [53] reported a diastereoselective domino oxa-Michael–Michael–Michael–aldol reaction as the key step for the construction of the hexahydro-6*H*-benzo[*c*]chromene skeleton of the biologically active natural product (+)-conicol (**26**).The protocol involved a tandem oxa-Michael–Michael reaction of 3-methyl-but-2-enal (**22**) and (*E*)-2-(2-nitrovinyl)-benzene-1,4-diol (**21**) followed by a domino Michael–aldol condensation with aldehyde **24** in the presence of TMS-protected diphenylprolinol **Ib**/AcOH as catalyst, affording the hexahydro-6*H*-benzo[*c*]chromene skeleton **25** (Scheme 13). The two reactions could be achieved in one pot, without the isolation of intermediate, in 55% overall yields.

#### 1.3. Reaction of 2-hydroxycinnamaldehydes/2-hydroxy-β-nitrostyrenes with α,β-unsaturated nitro compounds

**Scheme 14:**
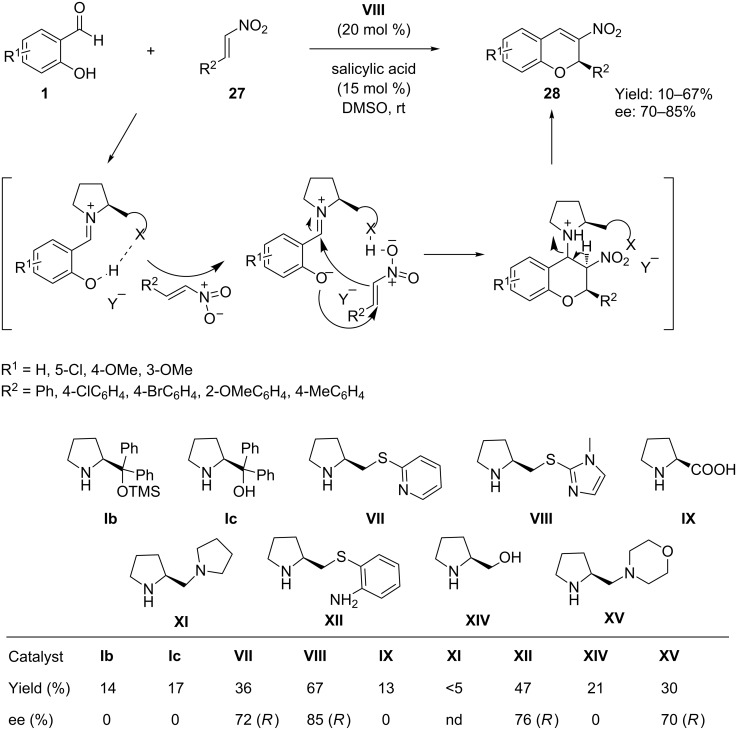
Tandem oxa-Michael–Henry reaction catalyzed by organocatalyst and salicylic acid, as reported by Xu.

Catalytic synthesis of nitrochromenes from salicylaldehydes and α,β-unsaturated nitro compounds are well documented in literature [54–59], but there are very few reports on the organocatalyzed synthesis of chiral nitrochromenes. Xu et al. [60] in 2008 first reported a novel organocatalytic tandem oxa-Michael–Henry reaction between salicylaldehydes **1** and nitroalkenes **27** employing (*S*)-1-methyl-2-(pyrrolidin-2-ylmethylthio)-1*H*-imidazole (**VIII**) as organocatalyst in the presence of salicylic acid as cocatalyst and in DMSO as solvent (Scheme 14). The reaction was interpreted as following an aromatic iminium activation strategy and provided 2*H*-nitrochromene derivatives **28** with moderate yields and moderate to good enantioselectivities. One of the limitations of this methodology is that only aromatic nitroalkenes tolerate the reaction conditions.

**Scheme 15:**
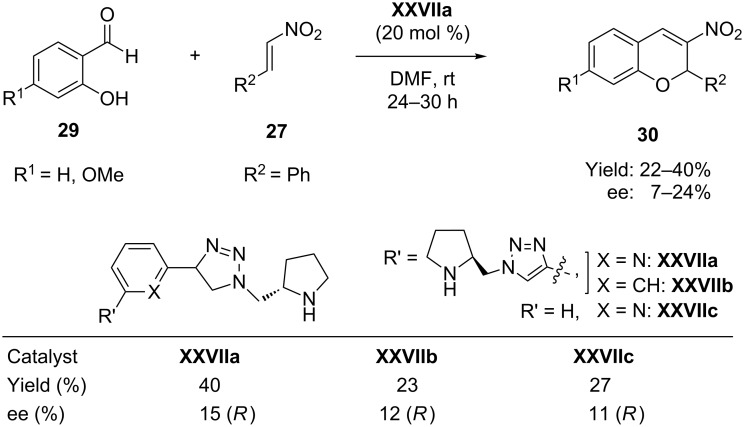
Asymmetric synthesis of nitrochromenes from salicylaldehydes and β-nitrostyrene, as reported by Sankararaman.

Subsequently a closely related reaction of salicylaldehydes **29** with β-nitrostyrene (**27**) employing pyrrolidine-triazole-based C2 symmetric organocatalysts **XXVIIa** was reported by Sankararaman et al*.* [61] for the asymmetric synthesis of nitrochromenes **30** (Scheme 15). The reaction gave poor enantioselectivities both in toluene (15% ee) and in DMF (24% ee).

**Scheme 16:**
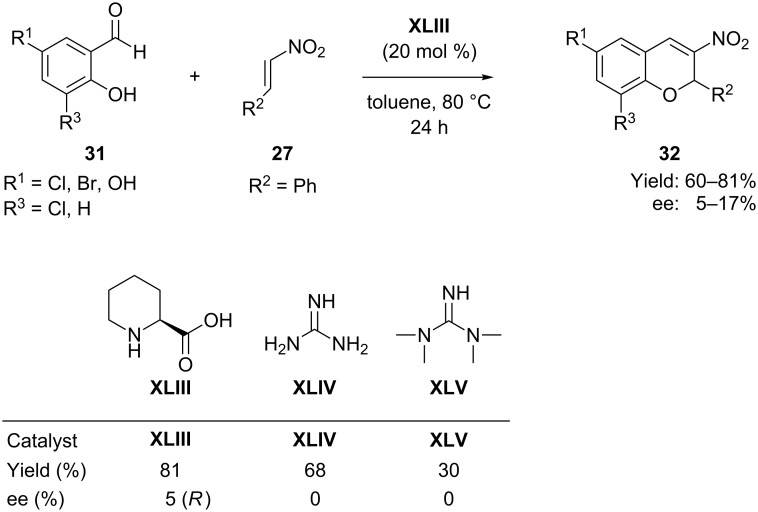
Domino Michael–aldol reaction between salicyaldehydes with β-nitrostyrene, as reported by Das and co-workers.

In 2010, Das et al. [62] reported an organocatalytic synthesis of 3-nitro-2-phenyl-2*H*-chromenes **32** without using any cocatalyst. The synthetic protocol involved an oxa-Michael–aldol reaction between salicylaldehydes **31** and β-nitrostyrene **27** in the presence of L-pipecolinic acid (**XLIII**) as organocatalyst in toluene at 80 °C, which proceeded with high yield but poor enantioselectivity (5–17% ee) (Scheme 16).

**Scheme 17:**
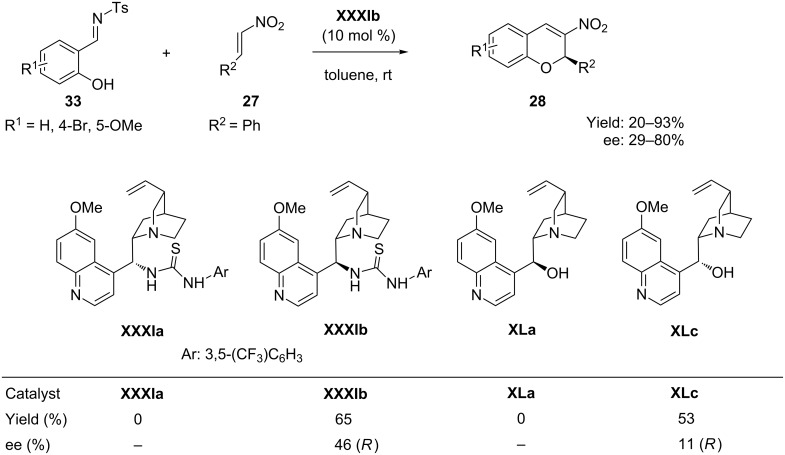
Enantioselective synthesis of 2-aryl-3-nitro-2*H*-chromenes, as reported by Schreiner.

Very recently, Schreiner et al. [63] reported a bifunctional thiourea **XXXIb** catalyzed tandem reaction of salicyl-*N*-tosylimines **33** with nitroolefins **27** in toluene at room temperature for the synthesis of nitrochromenes **28**, which resulted in poor yield and good enantioselectivities. A broad range of substituted nitrostyrenes was studied for these tandem *oxa*-Michel-aza-Henry-desulfonamidation processes to afford the corresponding 2-aryl-3-nitro-2*H*-chromenes by a kinetically controlled desulfonamidation step (Scheme 17).

### 2. Organocatalytic thio-Michael reactions to access functionalized thiochromenes

#### 2.1. Reactions of 2*-*mercaptobenzaldehydes with acyclic/cyclic α,β-unsaturated compounds

**Scheme 18:**
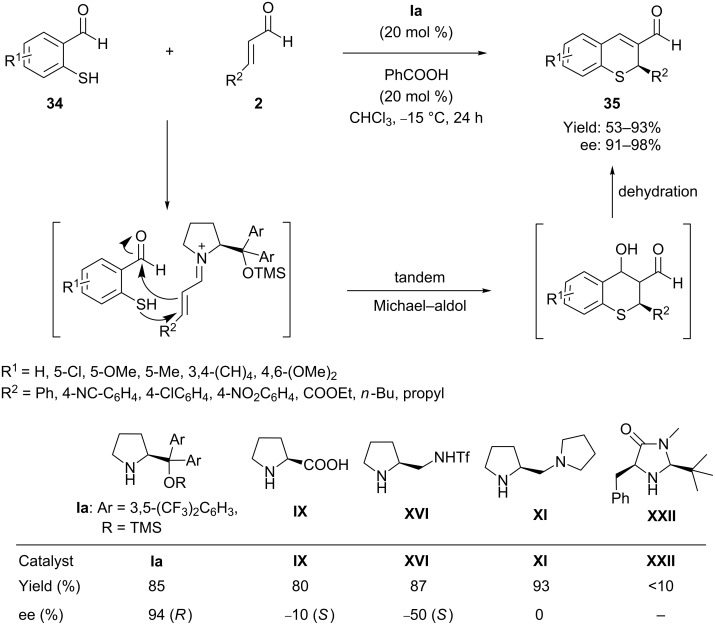
(*S*)-diphenylpyrrolinol silyl ether-promoted cascade thio*-*Michael–aldol reactions, as reported by Wang.

The organocatalytic enantioselective synthesis of chiral thiochromenes through tandem/domino-Michael addition reactions have featured in the literature of the past few years. In 2006, Wang et al. [64] first reported a very straightforward and effective method for the one-pot enantioselective synthesis of chiral 2*H*-thiochromene-3-carbaldehydes **35** through the reaction of 2-mercaptobenzaldehydes **34** with α,β-unsaturated aldehydes **2**, efficiently promoted by (*S*)-diphenylpyrrolinol silyl ether (**Ia**) as organocatalyst and benzoic acid as cocatalyst in toluene at room temperature (Scheme 18). The protocol provides good yield and high enantioselectivity of the desired products. The yield of the reactions does not reduce appreciably when substituents are present on the aromatic rings of the mercaptobenzaldehydes. Mechanistically, the reaction proceeds in a tandem thio-Michael–aldol reaction through the formation of active iminium species.

**Scheme 19:**
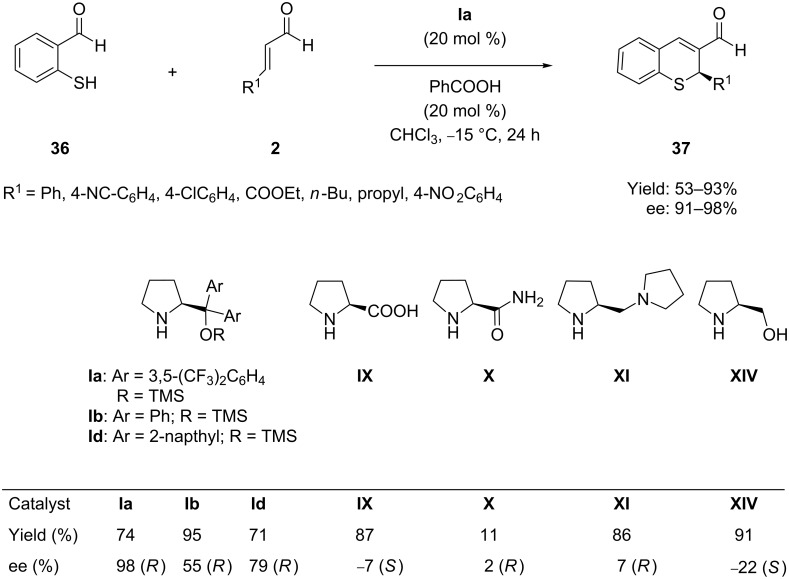
Organocatalytic asymmetric domino Michael–aldol condensation of mercaptobenzaldehyde and α,β-unsaturated aldehydes, as developed by Córdova.

Almost at the same time Córdova et al. [65] developed a similar protocol for enantioselective synthesis of pharmaceutically valuable 2*H*-1-benzothiopyrans **37** using the closely related catalyst **Ia** through the same iminium-enamine activation mode of the α,β*-*unsaturated aldehydes **2**. The asymmetric domino reactions proceeded with high yields (53–93%) and with excellent chemo- and stereoselectivities (up to 98% ee) in chloroform as solvent (Scheme 19). The analogous catalysts also show similar results under the same conditions after long reaction times.

**Scheme 20:**
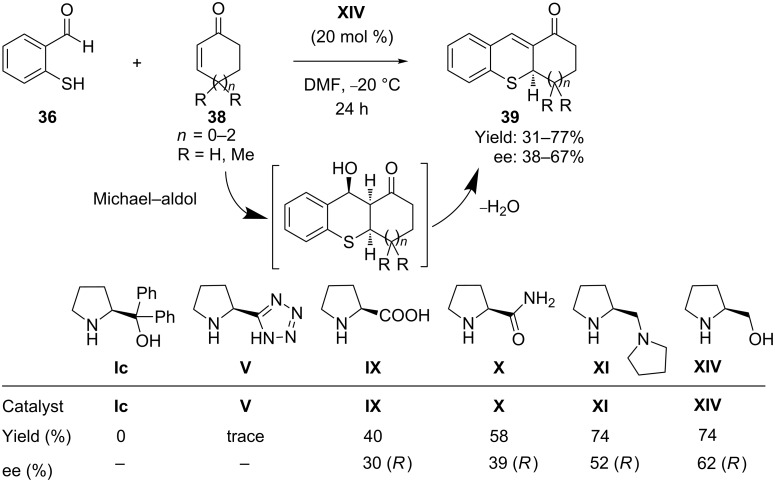
Organocatalytic asymmetric domino Michael–aldol condensation between mercaptobenzaldehyde and α,β-unsaturated cyclic ketones, as presented by Córdova and co-workers.

The same group [66] further presented a simple organocatalytic synthesis of tetrahydrothioxanthenones, derivatives of thiochromene. The catalytic domino thia-Michael–aldol reaction of 2-mercaptobenzaldehydes **36** and α,β-unsaturated cyclic ketones **38** proceeded in a highly chemoselective fashion, furnishing the corresponding products **39** in high yield and with moderate to good ee. The mechanism proposed involves the same iminium activation (Scheme 18) of the α,β-unsaturated cyclic ketones by the chiral pyrrolidine derivatives **XIV** (Scheme 20).

**Scheme 21:**
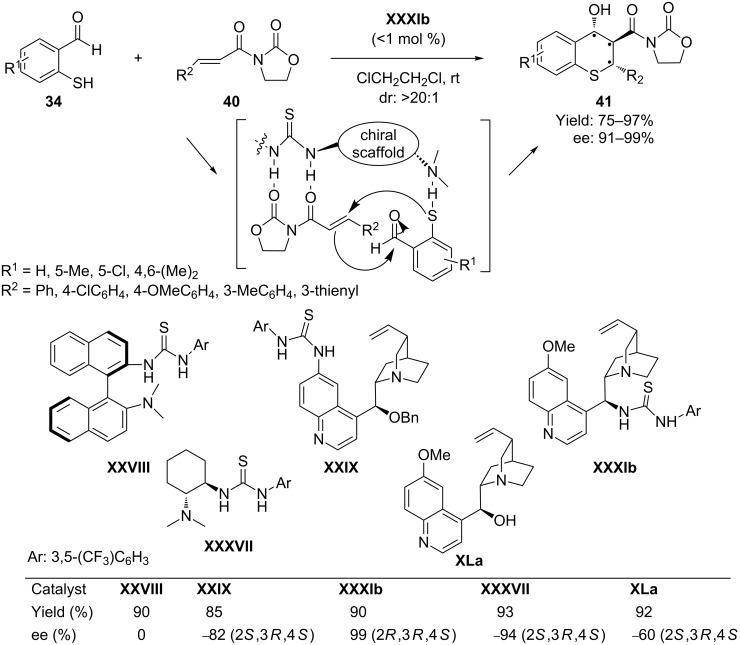
Hydrogen-bond-mediated Michael–aldol reaction of 2-mercaptobenzaldehyde with α,β-unsaturated oxazolidinones, as presented by Wang and co-workers.

In 2007 Wang et al. [67] presented a hydrogen-bond-mediated catalysis in order to perform highly enantio- and diastereoselective tandem Michael–aldol reactions for the synthesis of thiochromene derivatives. In a very low catalyst loading (<1 mol %) of hybrid thiourea-cinchona bifunctional chiral organocatalyst **XXXIb**, the reaction of 2-mercaptobenzaldehydes **34** and α,β-unsaturated oxazolidinones **40** by a synergistic noncovalent hydrogen-bonding dual-activation strategy afforded the highly chiral thiochromenes **41** with excellent yields and enantioselectivities (Scheme 21). The change of aldehyde group in the α,β-unsaturated system to a carboxylic acid derivative enables the process to be activated by hydrogen bonding rather than covalent interactions, which prevents the undesirable dehydration process.

**Scheme 22:**
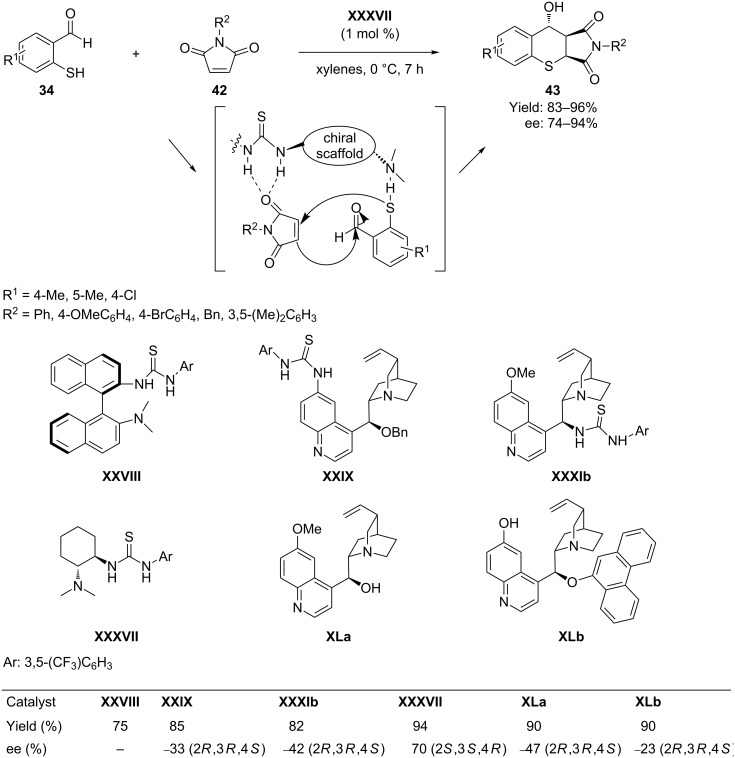
Domino Michael–aldol reaction of 2-mercaptobenzaldehydes with maleimides catalyzed by cinchona alkaloid thiourea, as reported by Wang’s group.

Subsequently, the same researchers [68] reported a synthesis of bioactive succinimide-containing benzothiochromenes by condensation of 2-mercaptobenzaldehydes **34** with maleimides **42** catalyzed by a bifunctional chiral amine thiourea **XXXVII**. Mechanistically, the reaction proceeded through a hydrogen-bond-mediated activation mechanism by using 1 mol % catalyst loading, which afforded versatile succinimide-containing benzothiopyrans **43** with the generation of three stereocenters in one single operation (Scheme 22). The method provides a general approach to the preparation of a range of substituted benzothiopyrans containing three stereocenters, with high enantiomeric excess (74–94% ee) and good to high levels of diastereoselectivities (3:1 to 20:1).

**Scheme 23:**
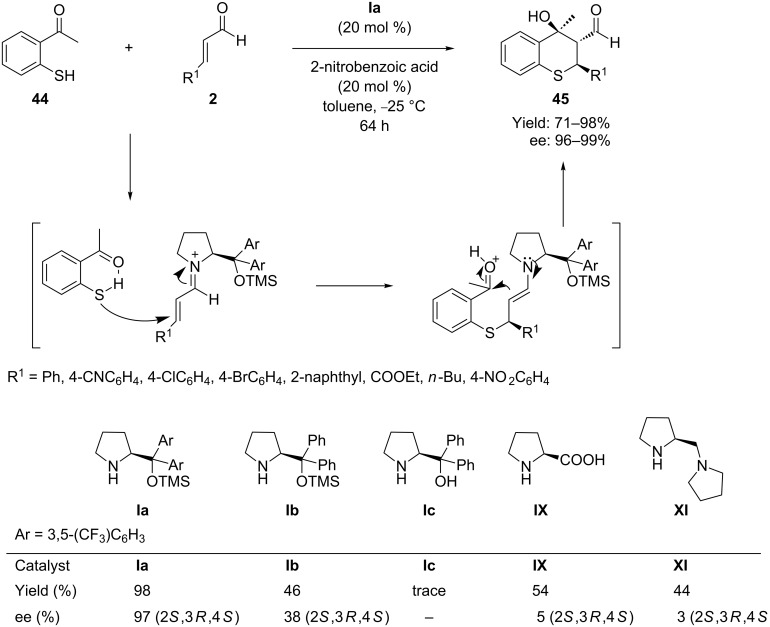
Domino thio-Michael–aldol reaction between 2-mercaptoacetophenone and enals developed by Córdova and co-workers.

In 2008 Córdova et al. [69] developed a novel tandem reaction between 2-mercaptoacetophenone **44** and α,β-unsaturated aldehydes **2** promoted by TMS-protected prolinol **Ia** as organocatalyst in the presence of 2-nitrobenzoic acid additive (Scheme 23). Avoiding the dehydration step, it is possible by this protocol to obtain thiochromans **45** bearing three contiguous stereocenters and a tertiary aldol structural motif with excellent enantioselectivities (96–99% ee) and yields (71–98%), and with good diastereocontrol (10:1 to 15:1 dr).

**Scheme 24:**
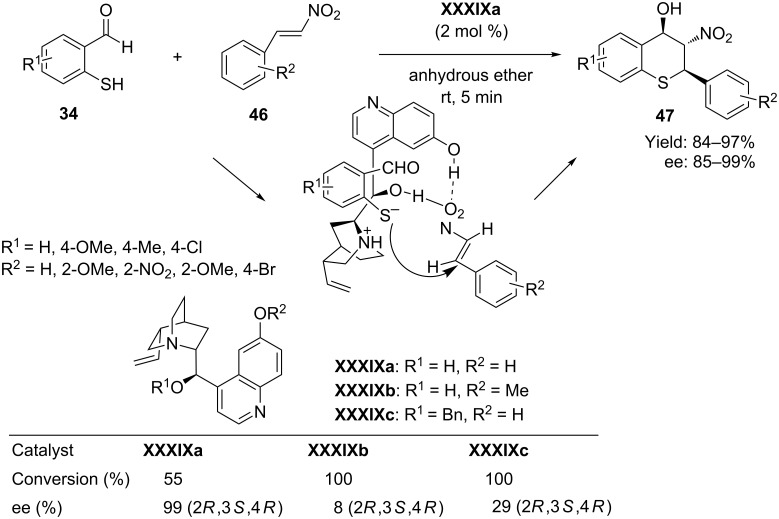
Enantioselective tandem Michael–Henry reaction of 2-mercaptobenzaldehyde with β-nitrostyrenes reported by Zhao.

In the same year Zhao et al. [70] reported an efficient synthesis of highly functionalized thiochromans having three chiral centers, using a tandem thio-Michael–Henry reaction of 2-mercaptobenzaldehydes **34** with β-nitrostyrenes **46** and using cupreine **XXXIXa** as catalyst in anhydrous diethyl ether. The protocol afforded the corresponding thiochroman **47** with excellent enantioselectivities and moderate diastereoselectivities (Scheme 24). A single recrystallization of the diastereomeric mixture from hexane/EtOAc enhances both the enantioselectivities (up to >99% ee) and diastereoselectivities (up to 98% de).

**Scheme 25:**
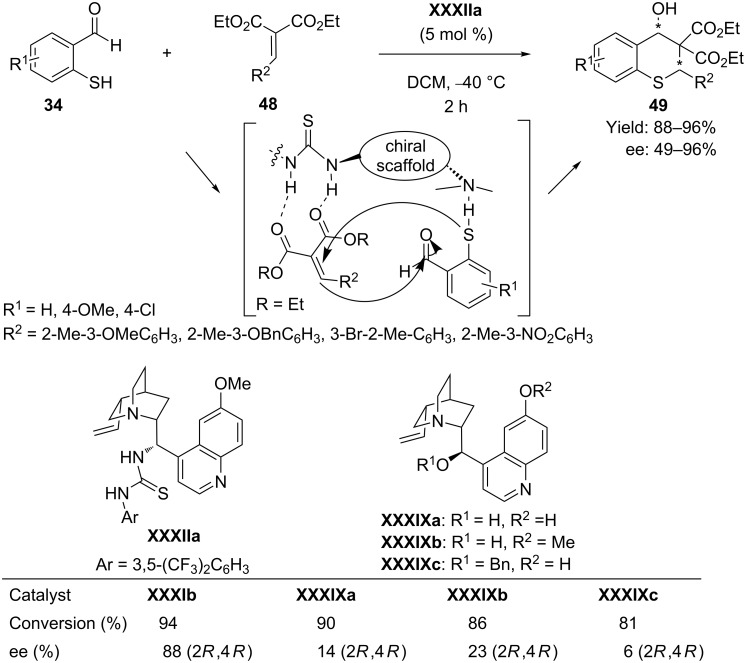
Enantioselective tandem Michael–Knoevenagel reaction between 2-mercaptobenzaldehydes and benzylidenemalonates, as developed by the Zhao group.

Later on the same group [71] developed another hydrogen-bond-mediated catalysis for the synthesis of tetrasubstituted thiochromans having three continuous stereocenters **49** following domino thio-Michael–Knoevenagel reaction between 2-mercaptobenzaldehydes **34** and benzylidenemalonates **48** using quinine thiourea **XXXIIa** as catalyst (Scheme 25). The steric and electronic environments of the substrates were found to affect profoundly the stereoselectivities of the reaction. It was observed that the diastereoselectivity of the reaction increased if there was a substituent at the ortho-position of the phenyl ring of the benzylidene moiety, while the enantioselectivity of the reaction decreased if the phenyl ring was substituted with an electron-withdrawing group. The best result, with yields up to 96% and enantioselectivities up to 96%, was obtained in DCM solvent at −40 °C.

**Scheme 26:**
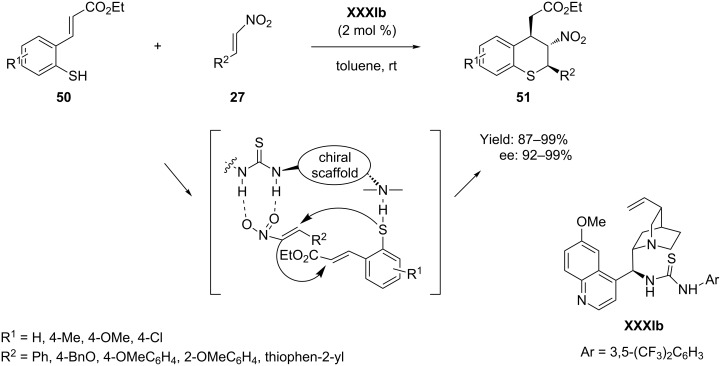
Cinchona alkaloid thiourea catalyzed Michael–Michael cascade reaction, as reported by Wang and co-workers.

An unprecedented asymmetric domino thio-Michael–Michael process, involving dynamic kinetic resolution, was reported by Wang et al. [72] using cinchona alkaloid amine-thiourea **XXXIb** as catalyst at a low catalytic loading of 2 mol %. Reaction of 3-(2-mercaptophenyl)-2-propenoic acid ethyl esters **50** with α,β-unsaturated nitro compounds **27** in toluene afforded chiral thiochromans **51** bearing three continuous stereogenic centers with high diastereo- and enantioselectivities by this novel cascade process (Scheme 26).

### 3. Organocatalytic aza-Michael reactions to access functionalized 1,2-dihydroquinolines

#### 3.1. Reaction of 2-aminobenzaldehydes with acyclic α,β-unsaturated compounds

**Scheme 27:**
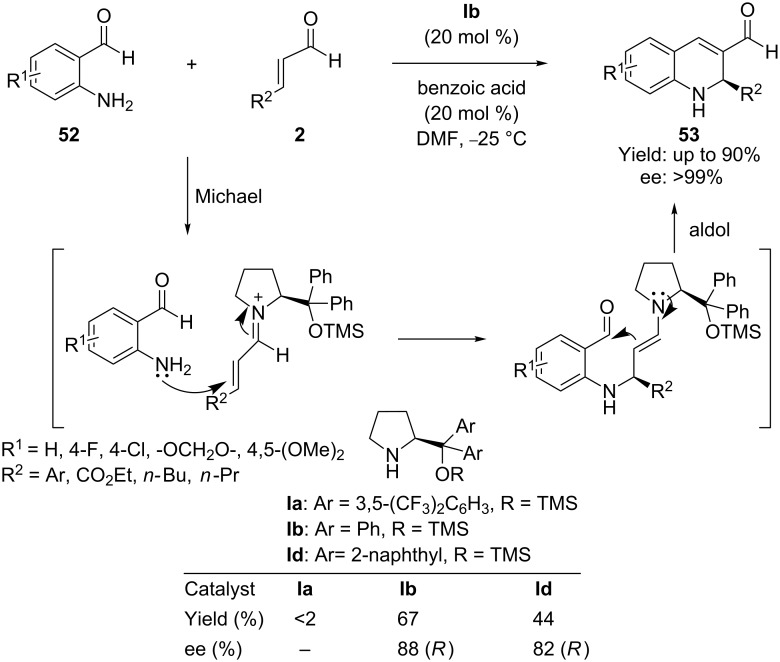
Domino aza-Michael–aldol reaction between 2-aminobenzaldehydes and α,β-unsaturated aldehydes, as reported by Córdova and co-workers.

The asymmetric organocatalytic Michael conjugate addition of an amine to an electron-deficient α,β-unsaturated system provides a unique reaction process because of the weaker nucleophilic character of the amine compared to a thiol or an alcohol. Córdova and co-workers [73] in 2007 first reported an asymmetric organocatalytic tandem aza-Michael–aldol reaction for the synthesis of 1,2-dihydroquinolines through the iminium activation strategy. Thus, the aza-Michael–aldol reaction between 2-aminobenzaldehydes **52** and enals **2** in the presence of diphenylprolinol trimethylsilyl ether **Ib** as catalyst and benzoic acid as additive, provides 1,2-dihyroquinolines **53** with high yields (90%) and high ee (>99%) (Scheme 27). Various enal substituents, such as aryl, alkyl and ester groups, are readily tolerated in the reaction. However, with β-alkyl α,β*-*unsaturated aldehydes the highest asymmetric induction was achieved in CH_3_CN without addition of an organic acid.

**Scheme 28:**
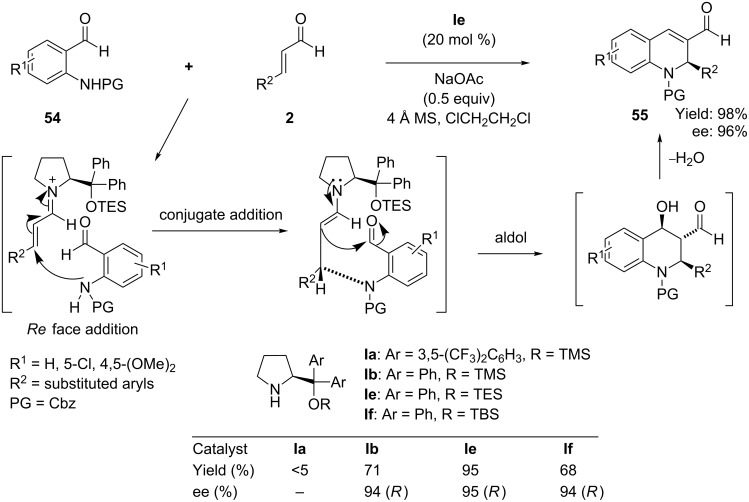
(*S*)-Diphenylprolinol TES ether-promoted aza-Michael–aldol cascade reaction, as developed by Wang’s group.

Subsequently, Wang and co-workers [74] reported a similar aza-Michael–aldol sequence employing more nucleophilic N-protected 2-aminobenzaldehydes in a basic medium as Michael donor for the construction of 1,2-dihydroquinoline derivatives. In their protocol, the reaction of 2-N-protected aminobenzaldehydes **54** with α,β-unsaturated aldehydes **2** promoted by (*S*)-diphenylprolinol TES ether **Ie** in the presence of NaOAc afforded the pharmaceutically valuable chiral 1,2-dihydroquinolines **55** with good yield (98%) and excellent ee (>96%) (Scheme 28). The reaction yield was dramatically improved in the presence of NaOAc and 4Å MS, without sacrificing enantioselectivity. The mechanistic study revealed that the conjugate-addition–aldol–dehydration sequence passes through the enamine intermediate. A wide range of readily available Michael acceptor α,β*-*unsaturated aldehydes are tolerated in the reaction. The protection group of the amino function also governs both reactivity and enantioselectivity. The Cbz-protected aminobenzaldehydes in dichloroethane afford the best results in a shorter reaction time.

**Scheme 29:**
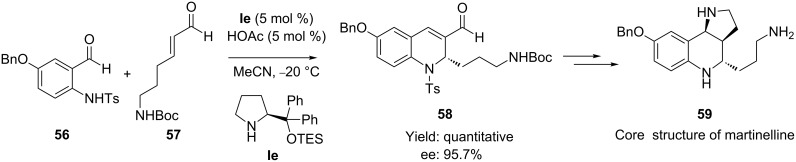
Domino aza-Michael–aldol reaction reported by Hamada.

By taking advantage of the above methodology, Hamada et al. [75] in 2008 reported a similar type of reaction for the construction of the 1,2-dihydroquinoline chiral core of martinelline (a nonpeptide bradykinin receptor antagonist) and its diastereoisomer. Using a domino aza-Michael–aldol reaction as the key step, reaction of aldehyde **56** with α,β-unsaturated aldehyde **57**, catalyzed by chiral prolinol ethers **Ie**/HOAc in acetonitrile, provided quinoline derivative **58** with high yield and with high enantioselectivity (Scheme 29). In contrary to the work of Li, the presence of 4Å MS had a negative effect on the yield, whereas addition of NaOAc did not alter the yield and enantioselectivity. On the other hand the addition of HOAc was found to enhance the catalytic activity of **Ie** by increasing the yield of reaction without the loss of enantioselectivity.

**Scheme 30:**
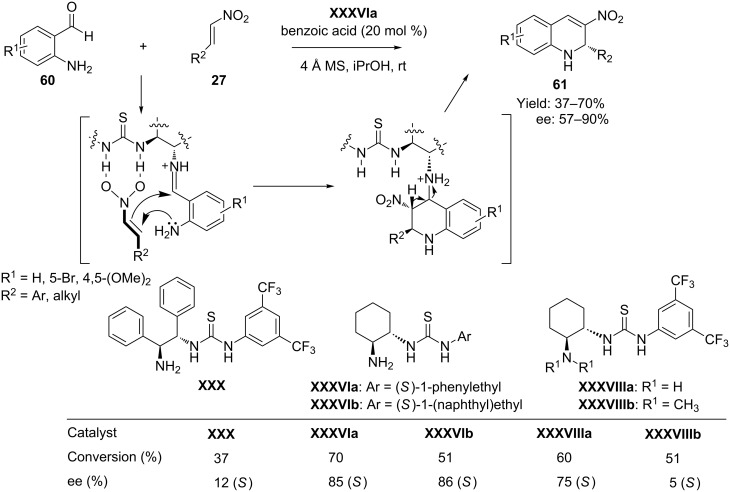
Organocatalytic asymmetric synthesis of 3-nitro-1,2-dihydroquinolines by a dual activation protocol reported by Xu and co-workers.

In 2009 Xu et al. [76] reported the first organocatalytic enantioselective domino aza-Michael–Henry reaction of 2-aminobenzaldehydes **54** and aromatic/aliphatic nitro olefins **27**, catalyzed by bifunctional thiourea catalyst **XXXVIa** in benzoic acid, to generate synthetically versatile 3-nitro-1,2-dihydroquinolines **61**. Synergistic activation of both reactants through stereoselective covalent activation and hydrogen-bond interaction allowed this transformation to take place under mild reaction conditions (propanol as solvent at room temperature) and afforded dihydronitroquinoline derivatives **61** with moderate yields and moderate to good enantioselectivities (Scheme 30).

**Scheme 31:**
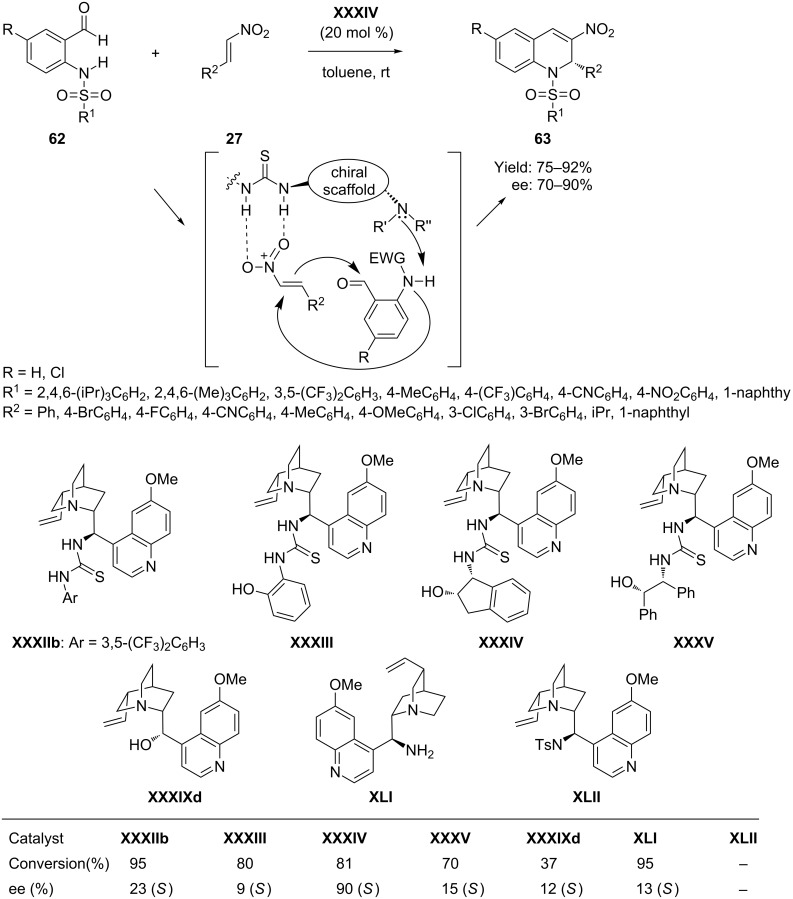
Asymmetric synthesis of 3-nitro-1,2-dihydroquinolines by cascade aza-Michael–Henry–dehydration reaction, as developed by Lu et al.

In 2010 a cascade aza-Michael–Henry dehydration process catalyzed by quinidine-derived tertiary amine-thiourea catalyst was developed by Lu et al. [77] for the one-step preparation of chiral 3-nitro-1,2-dihydroquinolines through the installation of suitable electron withdrawing groups at the amino function of aniline. Thus the condensation of N-protected aminobenzaldehydes **62** with substituted nitroolefins **27** mediated by tertiary amine-thiourea catalyst **XXXIV** in toluene at room temperature afforded 3-nitro-1,2-dihydroquinolines **63** in high yields (92%) and with high enantiomeric excess (90%) (Scheme 31). In this cascade reaction, the installation of electron-withdrawing groups on the amino moiety of 2-aminobenzaldehydes is anticipated to increase the aniline N–H acidity, the abstraction of which by the tertiary amine leads to an aza-Michael reaction. The thiourea group in the chiral catalyst is anticipated to have hydrogen-bonding interactions with the nitro group. The subsequent Henry reaction with the aldehydes, followed by dehydration, generated 3-nitro-1,2-dihydroquinolines.

## Conclusion

The advantage of organocatalysts in asymmetric synthesis has grown tremendously since its advent, and the power of tandem/domino/cascade-Michael addition reactions promoted by chiral organocatalysts has been intensely studied within this field. In this review, we have outlined some significant works concerning the organocatalytic enantioselective Michael addition reaction from three different points of view: The conjugate addition of hetero-centered nucleophiles to α,β-unsaturated compounds; in a more complex approach through tandem/domino/cascade-Michael reactions; and by using chiral amines as organocatalysts for the enantioselective synthesis of functionalized chiral chromenes, thiochromenes and 1,2-dihydroquinolines. Despite these impressive advances, there is plenty of room for new contributions and findings, particularly to the development of new organocatalysts that can enhance the reaction rate and enantioselectivity, and to improve the substrate scope such that unreactive Michael donors/acceptors may be used in these reactions under mild reaction conditions. Future work should be focused on the utilization of these powerful strategies for the efficient assembly of biologically interesting molecules, including natural products.
